# Dust sprinkling as an effective method for infecting layer chickens with wild‐type *Salmonella* Typhimurium and changes in host gut microbiota

**DOI:** 10.1111/1758-2229.13265

**Published:** 2024-05-15

**Authors:** Samiullah Khan, Andrea R. McWhorter, Daniel M. Andrews, Gregory J. Underwood, Robert J. Moore, Thi Thu Hao Van, Richard K. Gast, Kapil K. Chousalkar

**Affiliations:** ^1^ School of Animal and Veterinary Sciences The University of Adelaide Roseworthy South Australia Australia; ^2^ Bioproperties Pty Ltd Ringwood Victoria Australia; ^3^ School of Science RMIT University Bundoora Victoria Australia; ^4^ U.S. National Poultry Research Center USDA Agricultural Research Service Athens Georgia USA

## Abstract

Role of dust in *Salmonella* transmission on chicken farms is not well characterised. *Salmonella* Typhimurium (ST) infection of commercial layer chickens was investigated using a novel sprinkling method of chicken dust spiked with ST and the uptake compared to a conventional oral infection. While both inoculation methods resulted in colonisation of the intestines, the *Salmonella* load in liver samples was significantly higher at 7 dpi after exposing chicks to sprinkled dust compared to the oral infection group. Infection of chickens using the sprinkling method at a range of doses showed a threshold for colonisation of the gut and organs as low as 1000 CFU/g of dust. Caecal content microbiota analysis post‐challenge showed that the profiles of chickens infected by the sprinkling and oral routes were not significantly different; however, both challenges induced differences when compared to the uninfected negative controls. Overall, the study showed that dust sprinkling was an effective way to experimentally colonise chickens with *Salmonella* and alter the gut microbiota than oral gavage at levels as low as 1000 CFU/g dust. This infection model mimics the field scenario of *Salmonella* infection in poultry sheds. The model can be used for future challenge studies for effective *Salmonella* control.

## Graphical abstract

Dust sprinkling was an effective way to experimentally colonise layer chickens with *Salmonella* Typhimurium. *Salmonella* infection with 1000 CFU/g of dust altered the gut microbiota. This model mimics *Salmonella* infection in the field.

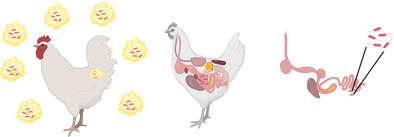

## INTRODUCTION

*Salmonella* belongs to the phylum Proteobacteria and is equipped with a type III secretory system that allows it to colonise the gastrointestinal tract of many animal species and humans. Intestinal colonisation and shedding into the environment create opportunity for ongoing faecal/oral transmission within a flock (Coburn et al., 2007). Once *Salmonella* has colonised the host gut, pathogenicity island 1 genes are activated during replication which encode for proteins that are instrumental in establishing the pathogen–host relationship (Mills et al., 1995). By eliciting host inflammation through the upregulation of invasion and virulence genes, *Salmonella* infection creates a metabolically favourable environment for the pathogen that results in improved replication in the intestine. The outcome of infection is dose‐ and serotype‐dependent. *Salmonella enterica* serovar Typhimurium is among the universal host serotypes that are associated with poultry production. *Salmonella* Typhimurium (ST) is one of the most commonly detected serovars in the poultry flock environment where it can colonise the gut of chickens and be shed in high concentration in the faeces. Horizontal transmission of ST is mainly considered to occur via the faecal‐oral pathway; however, infection of *Salmonella*‐contaminated dust via the respiratory route has been reported in turkeys and pigs (Harbaugh et al., 2006; Kallapura, Hernandez‐Velasco, et al., 2014; Oliveira et al., 2006). Initial colonisation of the intestinal lumen is followed by migration after invasion of gut epithelial cells, *Salmonella* disseminates into the internal organs through the bloodstream and can establish a systemic infection (Kallapura, Kogut, et al., 2014).

Clinical signs of salmonellosis in chickens are not always obvious, and the challenge of 1 week old chicks orally administered 10^3^ colony‐forming units (CFU) did not result in observable clinical signs of infection; however, pathological lesions were reported in the caeca upon dissection (Khan & Chousalkar, 2020a). *Salmonella* infection results in the activation of the gut mucosal immune response involved both in humoral and cellular pathways (Khan & Chousalkar, 2020a). In day‐old specific pathogen free chickens challenged through oral gavage with 10^8^ CFUs, ST could be quantified from caeca and liver 6 at 24 h post‐infection (p.i.), respectively (Withanage et al., 2004). The colonisation of the gut and internal invasion leads to continuous shedding and transmission within the flock.

The role of dust in the spread of *Salmonella* among flocks has been demonstrated previously (Gast et al., 1998; Holt et al., 1998). *Salmonella* has the potential to survive for at least a year in poultry‐associated dust (Davies & Wray, 1996). Detection of viable *Salmonella* in dust and drag‐swabs from nest boxes and egg belts shows the potential role of environmentally derived *Salmonella* in the horizontal transmission of this bacteria among the chickens within and between flocks (McWhorter & Chousalkar, 2019). However, the survival of *Salmonella* is also dependent upon the composition of the dust as well as physical parameters, such as water activity and total moisture level (McWhorter & Chousalkar, 2019). Moreover, *Salmonella* has the potential to survive better at 5°C when the moisture level is higher compared with 25 and 37°C (Zhang et al., 2020).

Layer gut microbiota plays an important role in host digestion. Several published reports demonstrate that *Salmonella* infection in layer chickens results in significant shifts in the composition of the gut microbiota (Huang et al., 2022; Kempf et al., 2020; Khan & Chousalkar, 2020b). For example, a significantly lower abundance of *Proteobacteria* and a shift in beta diversity were recorded in the caecum of *Salmonella* challenge chicks (Mon et al., 2015). Despite numerous studies on the effects of *Salmonella* on gut microbiota structure, it is not clear how route and infection dose affect gut microbiota composition in chickens.

There is abundant literature on ST challenge in chickens; however, different routes have not been widely investigated to establish which is more efficient in infecting birds. Additionally, the minimum threshold of ST required for the successful colonisation of the gut in layer chickens has not been well established. Therefore, the objective of this study was to compare oral versus sprinkled (aerosol) methods of ST dust challenge to establish the minimum threshold for infection. The hypothesis tested was that chickens can be infected with lower level of ST in the dust compared to the oral route. It was also hypothesised that different routes and infection levels might differentially affect the composition of the gut microbiota.

## EXPERIMENTAL PROCEDURES

Two separate in vivo experiments were performed to understand the role of the infection route and the minimum dose of wild‐type ST phage type 9, prepared in dust, for infecting layer chickens.

### Animal ethics approval

The Animal Ethics Committee at the University of Adelaide approved all chicken experimental work under the approval number S‐2020‐076. Animal Welfare Guidelines specified in the “Australian code for the care and use of animals for scientific purposes” were followed.

### *Hatching and rearing of* Salmonella *free layer chickens*

Fertile eggs of an Isa‐Brown parent flock were sourced from a local hatchery, incubated and hatched at the Roseworthy Campus of The University of Adelaide. The eggs were fumigated with formaldehyde gas prior to incubation to reduce exterior bacterial contamination. The chickens (mixed sex) were reared as per the management guide of Isa‐Brown International housed in custom‐designed pens containing chick paper. The chicks were supplied a commercial starter feed (Laucke Mill, South Australia) and untreated municipal water ad libitum throughout the study. This commercially prepared feed contained pre‐mixed lasalocid sodium as a coccidiostat but no other antibiotics. In Study 1, chickens were infected at 14 days of age, while in Study 2, chickens were infected at 21 days of age. There was a 1‐week age difference between Study 1 and Study 2 due to the coronavirus disease (COVID‐19) lockdown in South Australia. Due to the state‐wide lockdown, our Study 2 was delayed by a week. Infecting layer chickens at Weeks 2 and 3 was based on a previous study that had shown that newly placed chicks in a layer farm can become infected with *Salmonella* as early as Week 1 of age (McWhorter & Chousalkar, 2020).

### Salmonella *Typhimurium inoculum preparation in poultry‐associated dust*

Fine dust was scrapped from the top of the nesting boxes in a commercial free‐range egg production system using a sampling Whirl‐Pak® swab Speci‐Sponge®. The dust was autoclaved at 121°C for 15 min and dried in a dryer overnight at 45°C to rule out the chances of field contamination of dust with *Salmonella*. *Salmonella* free status of the autoclaved dust was confirmed by adding 1 g of dust sample to 9 mL of buffered peptone water and incubating overnight at 37°C. The incubated samples were processed for *Salmonella* isolation following the enrichment method previously described (McWhorter & Chousalkar, 2020). A pilot in vitro experiment was performed to determine the amount of water required to maintain the water activity of the autoclaved and overnight dried dust between 0.7 and 0.8 on a scale of 0–1. Water activity of a sample above 0.7 will keep microbes viable; however, *Salmonella* has been isolated from poultry dust with water activity as low as 0.53 (McWhorter & Chousalkar, 2019).

### Inoculum preparation and delivery for the route of infection study (Study 1)

A ST phage type 9 (PT9) strain (KC30) was revived on nutrient agar (ThermoFisher Scientific), subcultured in Luria Bertani (LB) broth (Oxoid), and the optical density (OD) was read at 600 nm to estimate the CFU/mL, with an OD600 of 1.0 estimated to be equivalent to 8 × 10^8^ CFU/mL. Bacterial culture (e.g., 10^6^ and 10^9^ CFU) was centrifuged at 5000 × *g* for 5 min, the supernatant discarded, and bacterial pellet was resuspended in 150 μL of 0.9% saline that was mixed with 1 g of dry and powdery dust (total weight 1.15 g). After through mixing, the inoculated dust was immediately used for the experiment. Dust containing 10^6^ CFU/g ST PT9 was sprinkled approximately 30 cm above the heads of 14‐day‐old chickens per group housed in a pen 55 cm wide × 125 cm long (6875 cm^2^). The floor of each pen was covered completely with chick paper so any of the sprinkled dust not inhaled by chicks settled on the paper and could be picked and or ingested by the chicks after application. For oral inoculation, individual chicks were administered approximately 1 mg out of the 1.15 g of dust spiked with 10^9^ CFU/g ST that equated to 10^6^ CFU/bird. The inoculation process involved withdrawing 1 mg of dust into the tip of a 3 mL syringe then delivered directly into the pharynx. Negative control treatment groups housed in a separate room received dust without ST.

### Inoculum preparation and delivery to assess the minimum infectious dose of the sprinkling infection model (Study 2)

ST PT9 (strain KC30) was revived on nutrient agar, subcultured in LB broth and the OD was read at 600 nm to estimate the CFU/mL. Based on the OD reading, the known culture volume was taken, which was equated to 1 × 10^6^ CFU. The samples were centrifuged at 5000 × *g* for 5 min, supernatant was discarded and the individual *Salmonella* pellet was resuspended in 150 μL of 0.9% saline. The 1 × 10^5^, 1 × 10^3^ and 1 × 10^1^ CFU/150 μL inoculum doses were prepared from serial dilutions of a stock *Salmonella* culture sample containing 1 × 10^6^ CFU/150 μL. This equated to 1 × 10^5^, 1 × 10^3^ and 1 × 10^1^ CFU/150 μL of saline. The 150 μL was added to 1 g of autoclaved dust. Therefore, ST PT9 inoculum was prepared at a dose of 10^1^, 10^3^ and 10^5^ CFU per gram of dust. Each gram of dust was sprinkled in a pen holding ten 21‐day‐old chickens, as explained above, and the control group received dust without *Salmonella*. An inoculum check was performed to confirm the CFU delivered to the chickens in the respective treatment groups.

### Detection of ST shedding through cloacal swabs

In both experiments, cloacal swabs were collected from individual chickens on Days 1, 3, 5 and 7 p.i. and processed for ST isolation through the culture enrichment method as previously described (Khan & Chousalkar, 2021). Cloacal swabs data were recorded as ‘1’ for *Salmonella* confirmed positive and ‘0’ for confirmed negative samples.

### S. *Typhimurium enumeration in organs through culture method*

From both experiments, at Day 7 p.i. 10 birds per treatment group were humanely euthanised, ST was quantified from spleen, liver and caecal tissues, using a culture method as previously described (Khan & Chousalkar, 2020a). Additionally, in the threshold of infection experiment (Study 2), lung samples were also collected for *Salmonella* enumeration. ST load was expressed as log_10_ CFU/g of tissue. From both the experiments, at Day 7 p.i., caecal contents were collected to investigate how the route of infection and or different doses of ST affected gut microbiota.

### Salmonella *quantification from caecal contents through quantitive polymerase chain reaction (qPCR)*

A ST PT9 (Strain KC30)‐specific qPCR that produces an 182 bp long amplicon was optimised using the primer pair (F: 5′‐TCTTTTTTCATCCCCACG‐3′ and R: 5′‐CGGTTTTACCACAAGCTAA‐3′) as described previously (McWhorter & Chousalkar, 2018). The primer pair was tested for specificity and amplification efficiency using 10‐fold serially diluted ST DNA. Quantitative qPCR was performed using a SensiFAST SYBR Hi‐ROX Kit (Bioline) in a 20 μL reaction volume. The reaction contained 10 μL SensiFAST buffer, 1 μL each of the forward and reverse primer (10 μM), 2 μL DNA template and 6 μL water. The cycling conditions in QuantStudio 6 (ThermoFisher Scientific) were: initial denature at 95°C for 3 min, 40 cycles of annealing at 60°C for 30 s and extension at 72°C for 30 s, a hold stage at 72°C for 5 min and melt from 60 to 95°C. The specificity of the primer pair was confirmed by a single peak detected in the melt curve analysis and running the amplicon on a 2% agarose gel. The amplification efficiency (%) was calculated as *E* = −1 + 10^(−1/slope)^.

### Salmonella *DNA fragment cloning and generation of standard curve*

A freshly generated qPCR product (182 bp amplicon length) of ST DNA was cloned into a plasmid (pCR4‐TOPO) that was inserted into DH5α‐T1^R^ chemically competent *Escherichia coli* cells as per the manufacturer's protocol of One‐Shot Chemical Transformation, TOPO TA Cloning Kit for Sequencing (Invitrogen). The recombinant plasmid was extracted using PureLink Quick Plasmid Miniprep Kit as per the manufacturer's protocol (Invitrogen). The insertion of the ST fragment into the plasmid was confirmed by qPCR, melt curve analysis and running the amplicon on 2% agarose gel. Wild‐type ST DNA was used as a positive control. The recombinant plasmid was serially diluted to construct a standard curve for the quantification of *Salmonella* load from caecal contents. The DNA copy number for the recombinant plasmid was calculated from the plasmid DNA concentration, as determined on a Nanodrop spectrophotometer (ThermoFisher Scientific), and the molecular weight of the plasmid with ST fragment insert.

### DNA extraction, 16S rRNA gene amplicon sequencing and downstream data analysis

Caecal content DNA was extracted from all the samples collected (*n* = 69) at the time of cull. The total DNA was extracted using a QIAamp Fast DNA Stool Mini Kit (Qiagen) following the modified protocol previously described (Khan & Chousalkar, 2021). DNA samples were sequenced for 16S rRNA gene amplicon microbiota profiling as previously described (Joat et al., 2023).

For the diversity profiling analysis (16S: 338F–806R [V3–V4]), dual indexing and variable spacer primers with forward (5′‐ACTCCTACGGGAGGCAGCAG‐3′) and reverse (5′‐GGACTACHVGGGTWTCTAAT‐3′) sequence primer pair were used (Fadrosh et al., 2014). The V3–V4 region of the 16S rRNA gene was amplified with Q5 high fidelity DNA polymerase (New England Biolabs). The cycling conditions for polymerase chain reaction were 98°C for 1 min, 35 cycles of 98°C for 10 s, 49°C for 30 s and 72°C for 30 s and final extension at 72°C for 10 min. The amplicon sequencing was performed using an Illumina MiSeq system (2 × 300 bp). The data were demultiplexed and quality filtered using Trimmomatic V0.39. A total of 2,477,368 reads were retained after filtering, with an average of 35,904 reads per sample. The microbiota analysis was performed in Quantitative Insights into Microbial Ecology 2 (QIIME2) (Bolyen et al., 2019). Quality filtering, denoising and chimaera removal were performed using DADA2 (Callahan et al., 2016) as a QIIME2 plugin with all recommended parameters. Taxonomy was assigned using SILVA v138.1 database (Quast et al., 2012) for the generation of the taxonomically assigned amplicon sequence variant (ASV) table. The ASV table was loaded into MicrobiomeAnalyst for data normalisation using the cumulative sum scaling option and the data were analysed using alpha diversity, beta diversity and Mann–Whitney/Kruskal–Wallis test options as per recommendation of the online software (Chong et al., 2020).

### Data analysis

The data obtained using the culture method (organ load and proportion of *Salmonella* positive and negative) and ST load in caecal contents by qPCR was analysed in GraphPad Prism 10 using one‐way analysis of variance and a non‐parametric test. Prior to data analysis, the data passed the normality test in GraphPad Prism. Level of significance was determined by Tukey's test at *p* < 0.05.

## RESULTS

### *Dust sprinkling is an effective method for infecting chickens with* Salmonella

**FIGURE 1:**
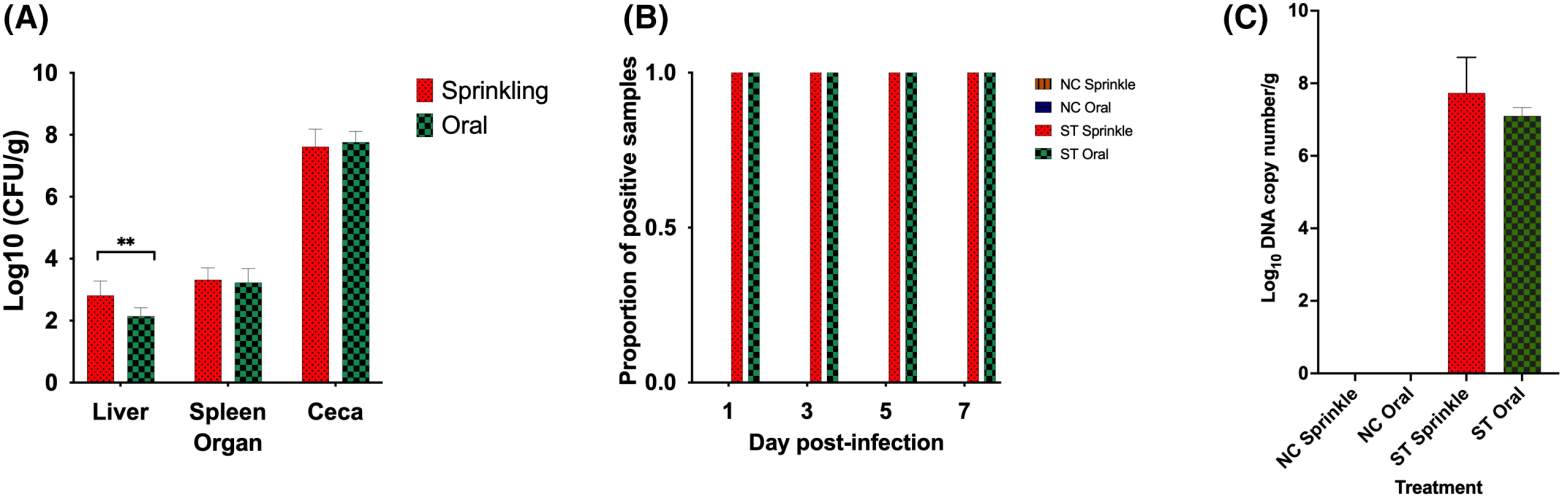
Study 1—*Salmonella* Typhimurium (ST) load and proportion of positive samples after inoculation with 10^6^ CFU either by sprinkling or oral inoculation of poultry dust. (A) Load of ST in different organs collected at Day 7 post‐infection (p.i.). (B) Proportion of positive cloacal swab samples of ST at Days 1, 3, 5 and 7 p.i. processed through the buffered peptone water and Rappaport–Vassiliadis–Soya broth enrichment steps. (C) ST load quantified through qPCR from caecal contents collected at Day 7 p.i. In this experiment, 14 days old layer chickens were challenged with ST inoculum prepared in dust and either sprinkled in a rearing pen or orally gavaged into individual chickens. Sprinkled and orally gavaged negative control treatment groups were included in the experiment. Organ samples for analysis were collected on Day 7 p.i. In the graphs, NC refers to negative control, while ST refers to *S*. Typhimurium PT9 infected groups. Values are mean ± standard error of mean. In each of the ST treatment groups, there were eight chickens, while in each of the negative control treatment groups, there were seven chickens.

Throughout the experimental period, the non‐infected control treatment group of chickens remained negative for ST. All infected chicks with either oral or sprinkling method, had an empty caeca. The level of ST quantified from the spleen and caeca of the sprinkled and orally infected chickens was not significantly different (Figure 1A). However, there was a significantly higher ST load in the liver of the sprinkle dust infected treatment group (Figure 1A). Both the sprinkle and oral administration of dust resulted in detectable ST in cloacal swabs after 24 h p.i. Cloacal swabs of all ST infected chickens were positive until the end of the experiment on Day 7 p.i. (Figure 1B). qPCR analysis showed that the levels of ST in the caeca were identical between sprinkle and oral challenged birds (Figure 1C).

### *Route of infection of* Salmonella *and changes in gut microbiota (Study 1)*

**FIGURE 2:**
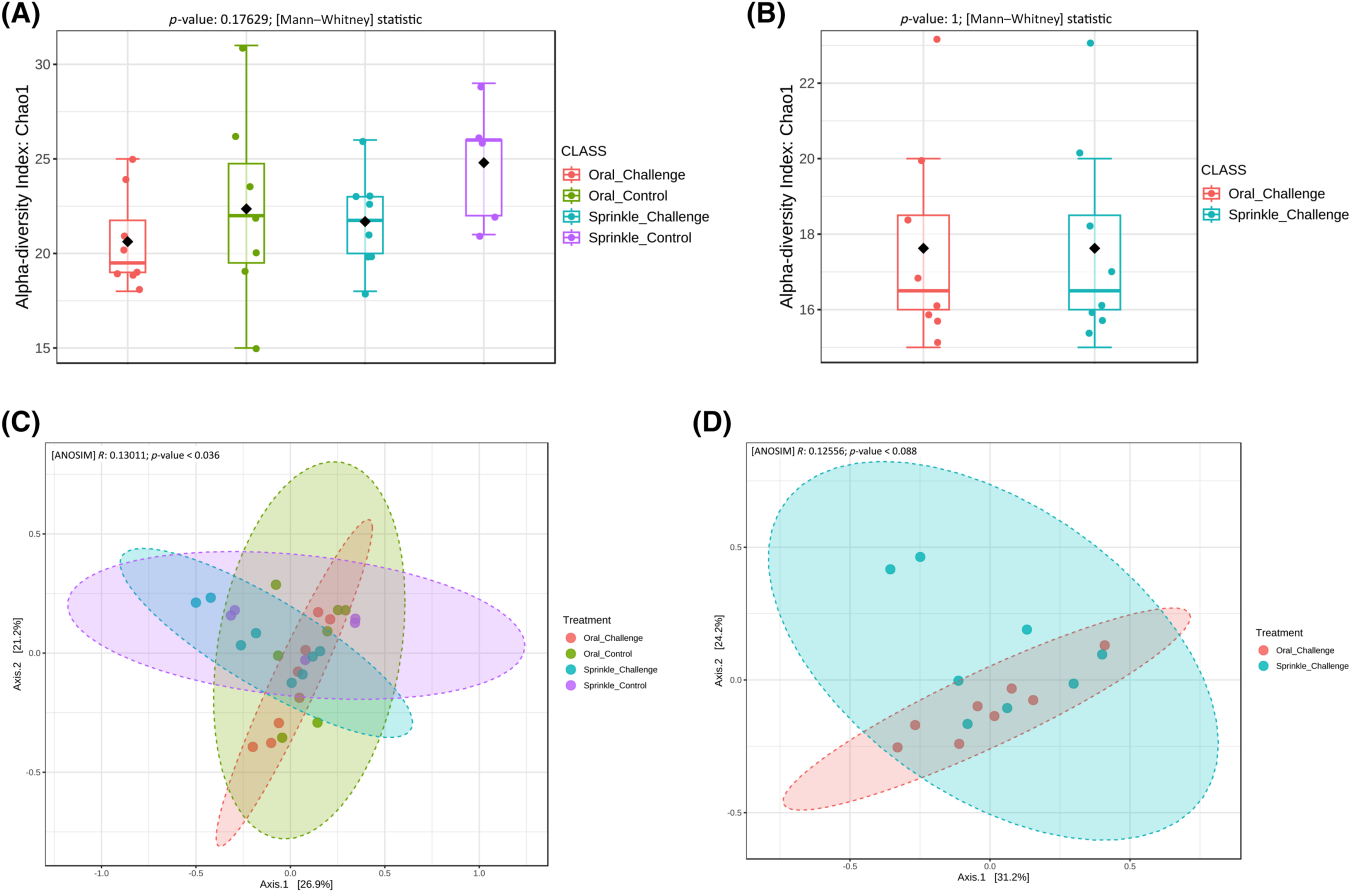
Study 1—Alpha and beta diversities of caecal microbiota affected by route of infection of *Salmonella* Typhimurium (ST) challenge. (A) Alpha diversity between the negative control and ST infected groups for each challenge method. (B) Alpha diversity between the sprinkled and oral challenged groups. (C) Beta diversity between the control and *S*. Typhimurium infected groups. (D) Beta diversity between the sprinkled and oral challenged groups.

Overall, the data showed that ST infection did not result in significant changes in the alpha diversity of the microbiota (Figure 2A). The alpha diversity was not significantly different between the sprinkle challenge and sprinkle control (*p* = 0.2939), and between the oral challenge and oral control (*p* = 0.7229) treatment groups. Also, the route of infection (sprinkle vs. oral) did not significantly (*p* = 1.000) change alpha diversity (Figure 2B). Beta diversity was significantly different (analysis of similarities [ANOSIM] *p* < 0.036) between the ST challenged and control groups (Figure 2C). The route of infection, oral versus sprinkle, did not significantly change the beta diversity of the caecal microbiota (Figure 2D).

**FIGURE 3:**
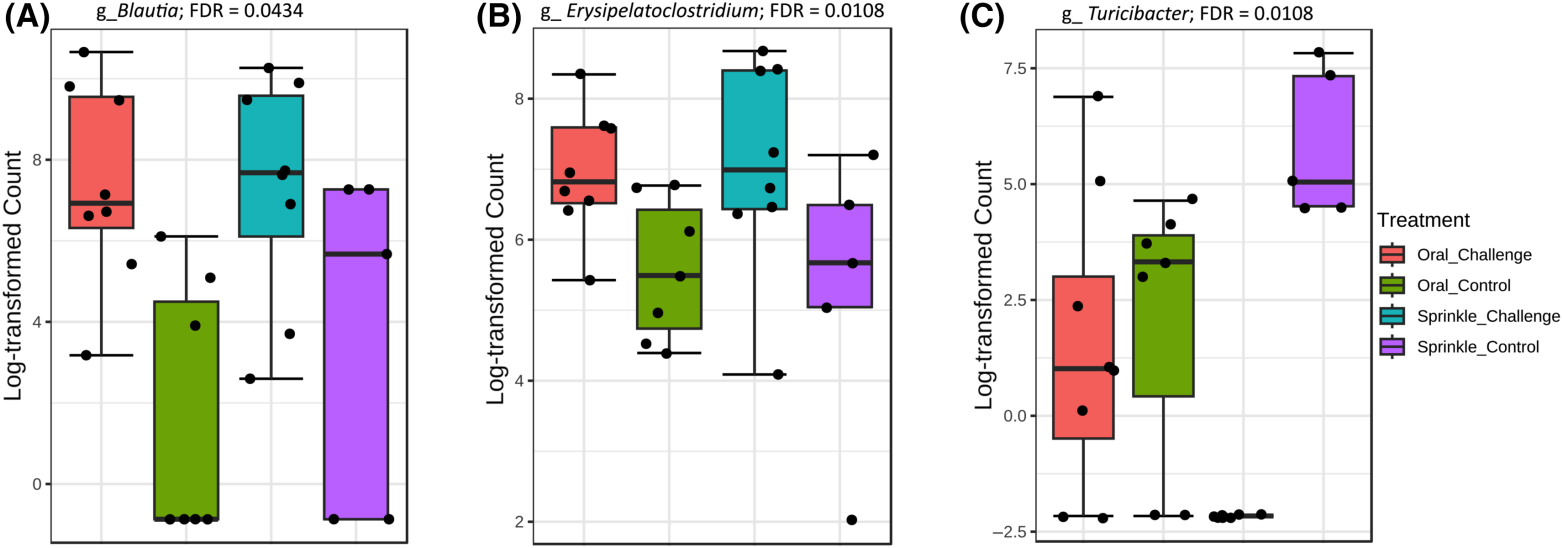
Study 1—Relative abundance of microbial genera affected by the route of infection of *Salmonella* Typhimurium challenge. Abundance level of (A) *Blautia*, (B) *Erysipelatoclostridium* and (C) *Turicibacter*. Amplicon sequence variant data were analysed at genus level in MicrobiomeAnalyst by using Mann–Whitney statistical analysis.

Route of infection of ST significantly affected (false discovery rate [FDR] <0.05) the relative abundance levels of *Blautia*, *Erysipelatoclostridium* and *Turicibacter* in the caeca of 2 weeks old chickens (Figure 3). Both *Blautia* and *Erysipelatoclostridium* relative abundance was significantly higher in the oral and sprinkle challenged treatment compared to the control groups (Figure 3A,B). The relative abundance levels of *Blautia* and *Erysipelatoclostridium* were not significantly different (FDR >0.05) between the oral and sprinkle treatment groups. The relative abundance level of *Turicibacter* was significantly lower in the sprinkle challenge compared with the oral challenge treatment group (Figure 3C). At the phylum level, there were relatively more *Firmicutes* in the sprinkle challenged group compared with the oral challenge group (Figure S1); however, the difference was not statistically significant (FDR = 0.0525).

### *Minimum infection dose required for* Salmonella *infection in chickens (Study 2)*

**FIGURE 4:**
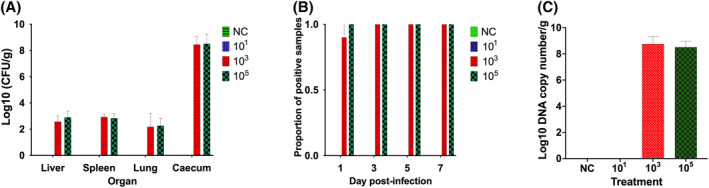
Study 2—*Salmonella* Typhimurium (ST) load in organs and proportion of positive cloacal swabs following infection by sprinkling spiked dust. (A) ST load in organs harvested at Day 7 post‐infection (p.i.). (B) Proportion of positive cloacal swab samples for ST collected at Days 1, 3, 5 and 7 p.i. (C) ST load quantified through qPCR from caecal contents collected at Day 7 p.i. In this experiment, ST inoculum was prepared using 10^1^, 10^3^ and 10^5^ CFU/g of dust and sprinkled in each pen of 21 days old layer chickens. Appropriate control group was included and organ samples for analysis were collected at Day 7 p.i. Values are mean ± standard error of mean. In each of the treatment groups, there were 10 chickens.

The inoculum check data showed that the treatment groups labelled as 10^1^, 10^3^ and 10^5^ CFU/g of dust contained 1.9 × 10^1^, 1.9 × 10^3^ and 1.9 × 10^5^ CFU/g of dust. The inoculum check data confirmed that the ST infected treatments received the right inoculum dose through the dust. Empty caeca were recorded in the treatment groups that received 10^3^ and 10^5^ CFU/pen of ST. In the threshold of infection experiment, no ST colonies were obtained from the negative control and the group that received 10^1^ CFU/g of dust sprinkled in a pen (Figure 4A). The ST load in the liver, spleen, lungs and caeca was not significantly different between the treatment groups that received 10^3^ and 10^5^ CFU per pen of chickens (Figure 4A). The average load of ST in the liver, spleen and lung samples was just over 2 log_10_, while in the caecal samples, the load was over 8 log_10_ irrespective of the infection doses of 10^3^ and 10^5^ CFUs. None of the cloacal swabs collected from the negative control and group that received 10^1^ CFU/pen were positive for ST after the culture enrichment method (Figure 4B). Ninety percent of cloacal swabs collected on Day 1 p.i. from the 10^3^ CFU treatment group were positive, while swabs collected on Days 3, 5 and 7 p.i. were 100% positive for ST. Cloacal swabs collected on Days 1, 3, 5 and 7 p.i. from 10^5^ CFU/pen treatment groups were 100% positive for ST (Figure 4B). Using qPCR, no ST was detected from the caecal contents collected on Day 7 p.i. from the negative control and the group that received 10^1^ CFU/pen of inoculum (Figure 4C). ST load quantified by qPCR from caecal contents from the 10^3^ and 10^5^ CFU/pen treatment groups was not significantly different (Figure 4C). The results indicate that the minimum infective dose, via the dust sprinkling method, is between 10^1^ and 10^3^ CFU/pen.

### S. *Typhimurium dosage and changes in gut microbiota*

**FIGURE 5:**
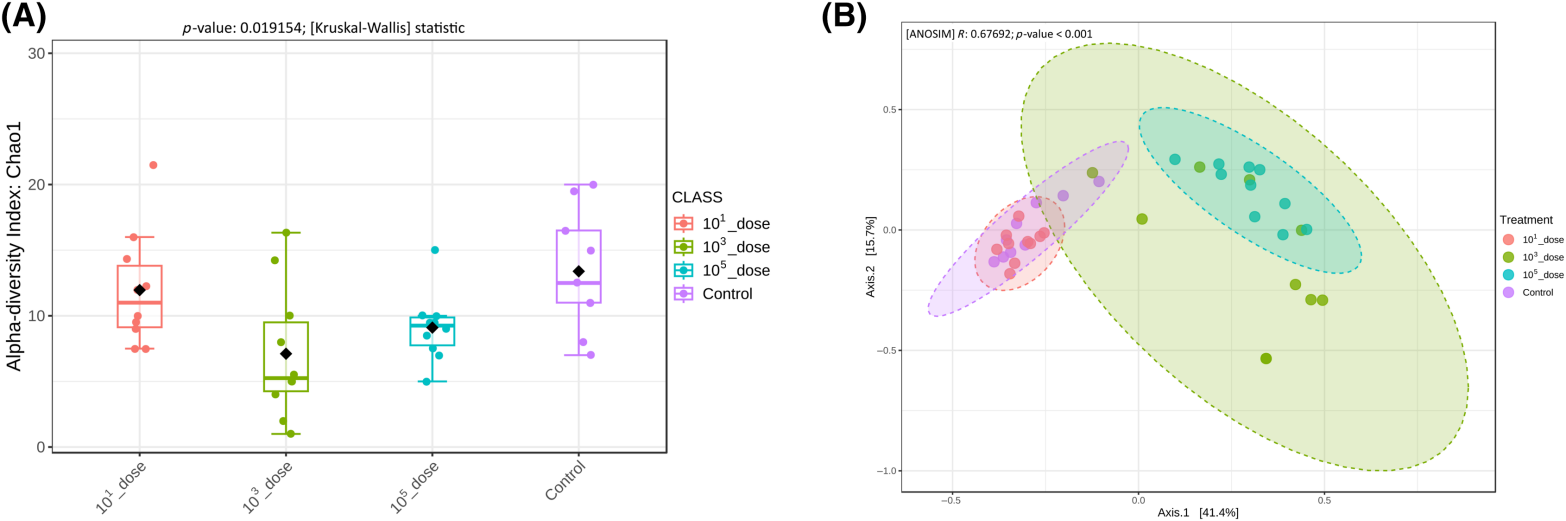
Diversities of caecal microbiota of layer chickens infected with different doses of *Salmonella* Typhimurium. Chickens were either challenged through dust model using different inoculum doses or left as control and sampled on Day 7 post‐infection for caecal contents DNA extraction. Both (A) Alpha diversity and (B) beta diversity indices were measured using MicrobiomeAnalyst.

Assessment of the effects of the infective dose of ST on the caecal gut microbiota, showed that both 10^3^ and 10^5^ CFU/pen significantly (*p* = 0.019154) decreased the alpha diversity of the gut microbiota (Figure 5A). The beta diversity of the 10^3^ and 10^5^ CFU/pen treatment groups was significantly dissimilar (ANOSIM *p* < 0.001) compared with the control and 10^1^ CFU/pen groups (Figure 5B). There was no significant difference in the beta diversity of the treatment groups that received 10^3^ and 10^5^ CFU/pen doses of ST through sprinkling of dust.

**FIGURE 6:**
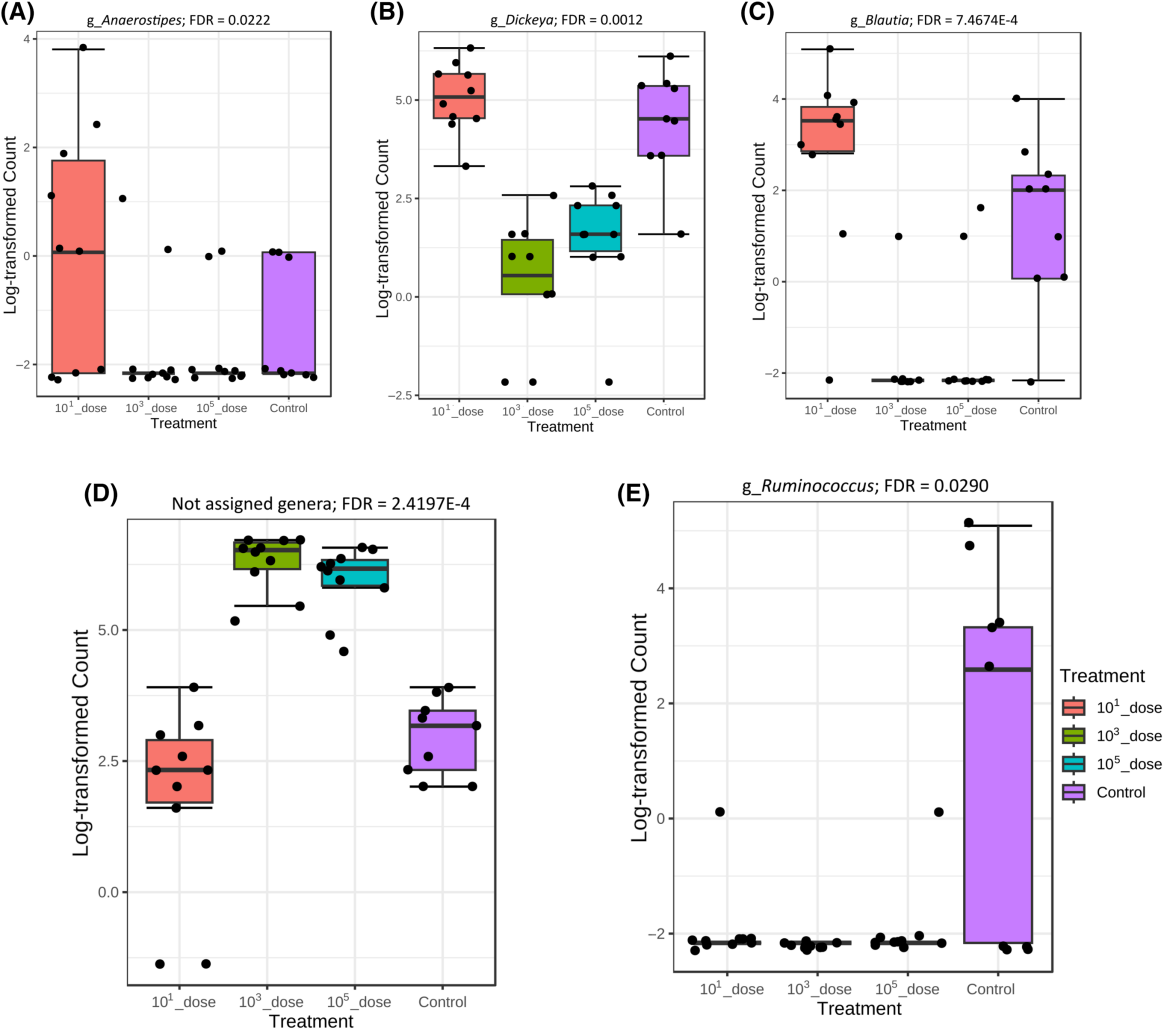
Effects of *S*. Typhimurium (ST) infectious dose on caecal microbiota in layer chickens. Relative abundance levels of genera (A) *Anaerostipes*; (B) *Dickeya*; (C) *Blautia*; (D) not assigned genera; and (E) *Ruminococcus*. Chickens in pens were challenged with 10^1^, 10^3^ or 10^5^ CFU of ST by the sprinkling dust method or left as uninfected controls. Caecal contents were collected at Day 7 post‐infection for microbiota analysis.

**FIGURE 7:**
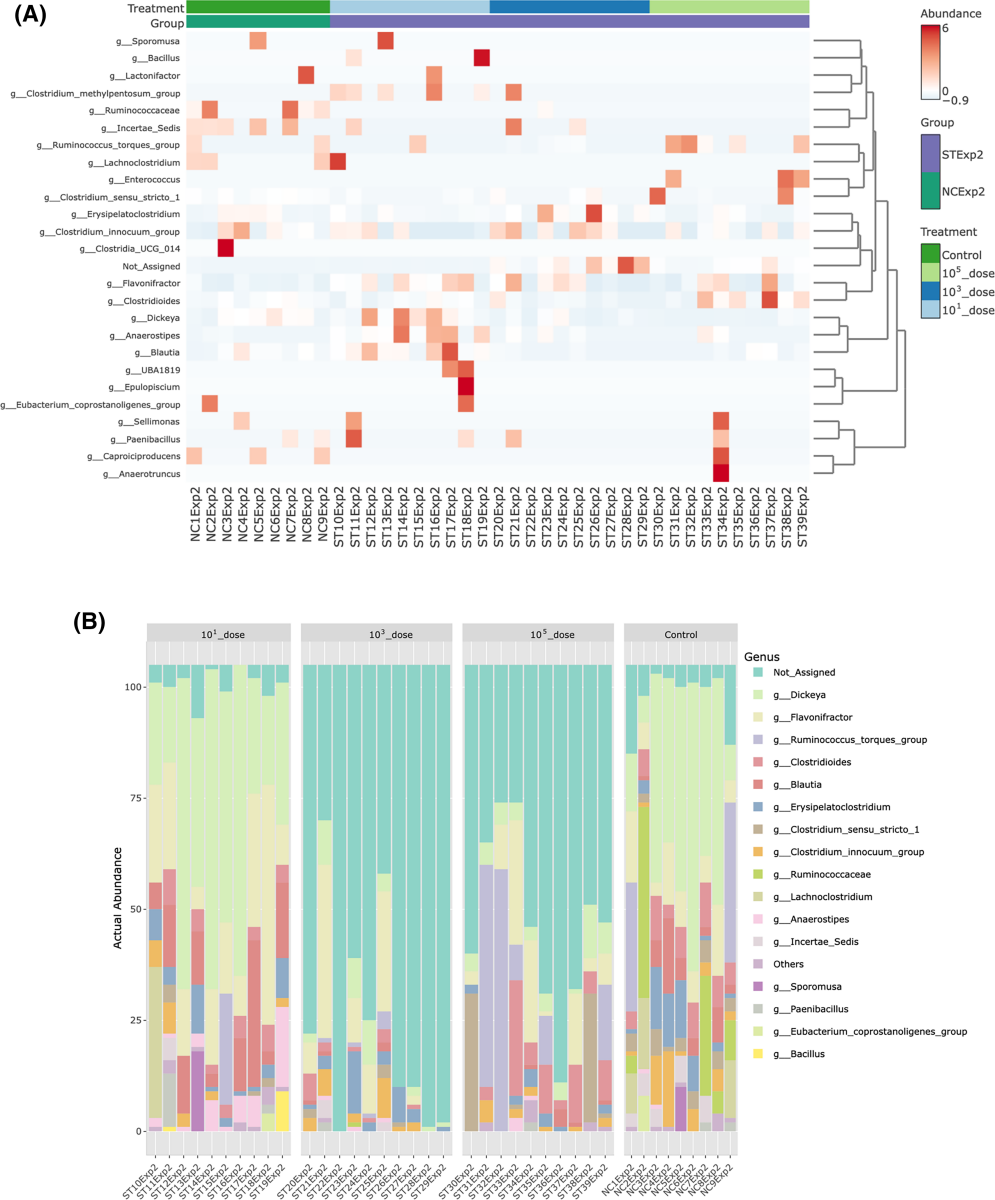
Visualised taxa of caecal microbiota affected by doses of *S*. Typhimurium infection. (A) Correlation heatmap showing taxa abundance. (B) Abundance profile of taxa. *X*‐axis of the panel graphs depicts individual samples.

The threshold of infection experiment data showed that ST infection resulted in a significantly lower (FDR <0.05) relative abundance of *Anaerostipes*, *Dickeya* and *Blautia* in the *Salmonella* 10^3^ and 10^5^ CFU per pen treatment groups compared with 10^1^ CFU/pen and control groups (Figure 6A–C). Interestingly, *Salmonella* challenge also increased the relative abundance of microbes that could not be assigned to genera based on the available reference taxonomy (Figure 6D). The relative abundance level of *Ruminococcus* was significantly lower in all the challenged groups compared with the control (Figure 6E). The correlation heatmap and taxa clustering graphs show that the structure of the caecal microbiota changed with the dose used for infection (Figure 7A,B).

## DISCUSSION

The main objective of the current study was to establish and test the effectiveness of the dust infection model in chickens to mimic field conditions. *Salmonella* has been persistently detected in dust collected from commercial cage and free‐range layer poultry farms (Gole et al., 2014; McWhorter & Chousalkar, 2019). Exposure to infected dust in the shed could result in infection after the introduction of chickens to the shed. There has been debate among the scientific community on the effectiveness of the route of infection and the minimum dose required for *Salmonella* challenge studies in chickens. Many *Salmonella* infection model studies in young layer chickens have administered an oral dose of either 10^8^ CFU (Gast, Guraya, Jones, Anderson, & Karcher, 2017; Gast, Guraya, Jones, Guard, et al., 2017; Mon et al., 2015) or 10^9^ CFU (Pande et al., 2016) per chicken, which are high and may not mimic doses typical encountered under field conditions. An in vitro study that assessed the transfer of ST from inoculated litter to the dust showed that increasing concentrations of bacteria in the litter resulted in a higher number of positive dust samples (Pal et al., 2021). Data obtained in the current study confirmed that dust could act as an effective carrier of ST and the sprinkling method resulted in a level of colonisation of layer chickens similar to that found when the oral route was used. Additionally, it was established that 19 (1.9 × 10^1^) CFU/g of dust were not sufficient to colonise the gut. However, it is worth to note that in field conditions, the infectivity of *Salmonella* in dust will also depend on its vegetative state, water activity and particle size of the dust. These findings have implications for the poultry industry and will prompt producers to pay more attention during farm decontamination and emphasises the importance of dust removal during regular farm cleaning.

In the current study, the data from the experiment that tested the effectiveness of the route of infection showed that dust spiked with 10^6^ CFU infected 100% of 2‐week‐old chicks when delivered by both the sprinkling and oral routes. However, the dust sprinkling route was effective, as it resulted in significantly higher colonisation of liver, confirming the effectiveness of this method. It is worth noting that the sprinkled dust would have been inhaled and orally picked up from the pen floor as well due to the inherent picking nature of avian species. This behaviour mimics field conditions if accumulating dust contains viable *Salmonella*. Chickens in the surrounding environment can pick it up, especially in the shed environment. Despite this inherent difference in the route of infection, the sprinkle method treatment group showed the same level of colonisation in caeca and invasion into spleen.

With the minimum infection dose when inoculated by sprinkling dust (Study 2), *Salmonella* was not isolated by direct plating and culture methods from the control and the group that received 1.9 × 10^1^ CFU/g of dust per pen (approximately 2 CFU/bird). This depicts that 19 CFU were not sufficient to establish infection in ten chickens housed in a single pen (687.5 cm^2^). It is worth to note that fewer cells of *Salmonella* may not survive in the upper segments of the chicken gut due to the low pH environment. Previous studies showed that intra‐cloacal administration of 2 CFU of ST were sufficient to cause infection in day‐old chickens (Cox et al., 1990), noting that intra‐cloacal administration was direct inoculation method in the host. A 100‐fold higher dose of *Salmonella* was required to colonise in adult birds via oral route compared with intra‐cloacal route (Cox et al., 1990; Leaney et al., 1978)., which could be due to low pH in the upper segments of the gut. Broiler chickens challenged with 10^6^ CFUs of ST or Enteritidis through intra‐tracheal route established colonisation more effectively in the caeca and invasion into liver and spleen compared with the oral route (Kallapura, Hernandez‐Velasco, et al., 2014). Data obtained in the current study showed that sprinkling 1 g of dust carrying ST was sufficient to colonise the gut and invaded into spleen and liver of 21 days old layer chickens. In the current study, the higher inoculum dose (10^5^ CFU/g of dust per pen) did not result in significantly higher load in the liver, spleen and caeca compared with lower inoculum dose (10^3^ CFU/g of dust per pen). The non‐significant difference in the load of ST in caeca on Day 7 p.i. between the 10^3^ and 10^5^ CFU/g of dust treatment groups shows its level of replication with the gut. In a shed environment, turkeys exposed to *Salmonella*‐contaminated litter (2.6 × 10^5^ CFU/g) for 2 h developed the infection (Harbaugh et al., 2006). In the current study, both the route and threshold of infection experiments confirmed that dust carrying ST was effective in colonising layer chickens.

The 16S rRNA gene amplicon sequence data obtained in both the infection model experiments showed that ST altered the composition of caecal microbiota. In Study 1, ST did not result in significant changes in the alpha diversity between the treatment groups. However, in Study 2, alpha diversity was significantly affected by ST challenge. This difference between the two studies could not be explained as housing, diet and husbandry conditions remained the same. The only differences were inoculum dose and time of ST challenge. In Study 1, the ST challenge was introduced at the 2 weeks of chicken age, while in Study 2, chickens were 3 weeks old at the time of challenge. In Study 1, the inoculum dose was 10^6^ CFU/g of dust, while in Study 2, the doses were 10^1^, 10^3^ and 10^5^ CFU/g of dust. Future studies should focus on whole‐genome sequencing of microbiota with large sample sizes to determine the relationship between alpha diversity and *Salmonella* challenge. Both the oral and sprinkle methods of infection resulted in significantly dissimilar beta diversity of the caecal microbiota. The non‐significant difference in the beta diversity of the oral and sprinkle treatment groups showed that once ST is introduced into the gut, it has the potential to establish a niche and change the structure of microbial communities. The threshold of infection experiment showed that infection dose of 10^3^ CFU/ pen was sufficient to change microbial communities structure. A non‐significant difference between the 10^1^ CFU and control groups for the diversities and abundance of caecal microbial communities was due to the exposure of the chickens to very low level (19 viable CFU/pen) of ST during challenge, which was not sufficient to establish gut colonisation for active shedding of ST in the faeces. The 16S data analysed for abundance of microbial communities resulted in lower relative abundance levels of *Blautia*, *Dickeya*, *Erysipelatoclostridium*, *Anaerostipes*, *Turicibacter* and *Ruminococcus* showed that these microbial genera were reduced as a result of ST infection. The *Blautia* abundance in infected groups was significantly higher in Study 1 than in Study 2. This difference could be attributed to different doses of ST in Study 2. Additionally, the chicken age in Study 2 at *Salmonella* challenge time was 3 weeks, while in Study 1, chickens were 2 weeks old. In our previous study, the ST challenge decreased the abundance of *Blautia* in the chicken gut (Khan & Chousalkar, 2020b). In *S*. Enteritidis challenged chickens, the abundance of *Blautia* initially decreased but then increased in the gut (Liu et al., 2018). Based on relevant literature, whole‐genome sequencing analysis of gut microbiota is suggested to determine the true relationship of the *Blautia* population with ST. An important caveat of gut microbiota analysis is that the gut microbiota can vary significantly despite birds being from the same genetic line, originating from the same hatchery, housed in the same animal facilities and given the same feed (Rychlik, 2020). As reviewed in (Liu et al., 2021), many species of *Blautia* utilise a range of carbohydrates including mannitol, lactose, maltose and cellobiose; however, its more specific role is the production of acetate through the expression of acetyl‐CoA carboxylase (Polansky et al., 2016). *Dickeya* falls in *Pectobacteriaceae*, and it has been shown to be the most abundant genera in the gut of layer chickens (Joat et al., 2023). *Turicibacter*, present in the gut of chickens (Rychlik, 2020; Siegerstetter et al., 2018), has previously been isolated from chicken eggshell (Maki & Looft, 2022); however, its wider functions in the gut are yet to be studied. The abundance of *Turicibacter* was increased in the gut of broilers fed with supplemented diet containing dried distillers grains with solubles (Abudabos et al., 2017). *Ruminococcus* are butyrate producers, and have been shown to be highly abundant in chicken caecum (Saxena et al., 2016; Vlasatikova et al., 2024). As shown in the broiler chickens gut, *Anaerostipes* species produce butyrate (Eeckhaut et al., 2010), which is a preferred source of energy for intestinal epithelial cells. In the current study, the lower abundance levels of the above‐mentioned genera in the caecal lumen of the challenged chickens show the dysbiosis caused by ST infection.

## CONCLUSIONS

Dust sprinkling was an effective ST challenge model in experimental settings. Irrespective of the route of infection and dose (10^3^ or 10^5^ CFU), ST was detectable through cloacal swabs after 24 h p.i. Irrespective of the route and inoculum dose (10^3^ or 10^5^ CFU), *Salmonella* resulted in changes in gut microbiota that were significantly different from the control groups. The study has field implications by suggesting that dust in poultry sheds will act as a potential source of ST for cross contaminating nearby sheds.

## AUTHOR CONTRIBUTIONS

**Samiullah Khan:** Data curation (equal); formal analysis (lead); investigation (supporting); methodology (lead); validation (lead); writing – original draft (lead). **Andrea R. McWhorter:** Conceptualization (equal); data curation (equal); investigation (equal); project administration (equal); writing – review and editing (equal). **Daniel M. Andrews:** Conceptualization (equal); investigation (equal); project administration (equal); writing – review and editing (equal). **Gregory J. Underwood:** Conceptualization (equal); project administration (equal); supervision (equal); writing – review and editing (equal). **Robert J. Moore:** Formal analysis (equal); investigation (equal); software (equal); writing – review and editing (equal). **Thi Thu Hao Van:** Data curation (equal); formal analysis (equal); writing – review and editing (equal). **Richard K. Gast:** Conceptualization (equal); project administration (equal); writing – review and editing (equal). **Kapil K. Chousalkar:** Conceptualization (lead); data curation (lead); investigation (equal); project administration (lead); resources (lead); supervision (lead); writing – review and editing (lead).

## CONFLICT OF INTEREST STATEMENT

Dr. Gregory J. Underwood and Dr. Daniel M. Andrews are employed by Bioproperties Pty Ltd, Australia. All other authors declare no conflict of interest.

## Supporting information


**Figure S1.** The comparison of the sprinkle challenged with the oral challenge group.

## Data Availability

The 16S rRNA sequence data are available from the Sequence Read Archive (SRA) database in NCBI under the accession number PRJNA1038781.
